# Leveraging the placenta to advance neonatal care

**DOI:** 10.3389/fped.2023.1174174

**Published:** 2023-05-15

**Authors:** Karen K. Mestan, Sandra L. Leibel, Eniko Sajti, Betty Pham, Samantha Hietalati, Louise Laurent, Mana Parast

**Affiliations:** ^1^ Department of Pediatrics/Division of Neonatology, University of California, San Diego School of Medicine, La Jolla, CA, USA; ^2^ Department of Pediatrics/Division of Neonatology, Rady Children's Hospital of San Diego, San Diego, CA, USA; ^3^ Department of Obstetrics, Gynecology and Reproductive Sciences/Division of Maternal Fetal Medicine, University of California, San Diego School of Medicine, La Jolla, CA, USA; ^4^ Sanford Consortium for Regenerative Medicine, La Jolla, CA, USA; ^5^ Department of Pathology, University of California, San Diego School of Medicine, La Jolla, CA, USA

**Keywords:** neonatal intensive care unit (NICU), placental pathology, prematurity and low birth weight, preeclampsia, bronchopulmonary dysplasia (BPD), chorioamnionitis, neonatal outcome

## Abstract

The impact of placental dysfunction and placental injury on the fetus and newborn infant has become a topic of growing interest in neonatal disease research. However, the use of placental pathology in directing or influencing neonatal clinical management continues to be limited for a wide range of reasons, some of which are historical and thus easily overcome today. In this review, we summarize the most recent literature linking placental function to neonatal outcomes, focusing on clinical placental pathology findings and the most common neonatal diagnoses that have been associated with placental dysfunction. We discuss how recent technological advances in neonatal and perinatal medicine may allow us to make a paradigm shift, in which valuable information provided by the placenta could be used to guide neonatal management more effectively, and to ultimately enhance neonatal care in order to improve our patient outcomes. We propose new avenues of clinical management in which the placenta could serve as a diagnostic tool toward more personalized neonatal intensive care unit management.

## Introduction

The placenta is the transient organ of pregnancy that plays an intricate role in the growth, development, and protection of the fetus. Historically, research on the placenta has been predominantly focused on the role of maternal health on placental development and function, as well as the downstream effects on pregnancy health, fetal development, and the transition to neonatal life. However, there has been a relative paucity of guidance aimed at applying knowledge about placental pathology to improve the management of critically ill newborn infants, in particular, premature infants, who may spend the first few weeks to months in the neonatal intensive care unit (NICU). Barriers to using the placenta to guide neonatal management include the relative lack of understanding among neonatologists about what certain placental findings may indicate about their patients and the lack of robust workflows to facilitate education, communication, and information transfer among obstetricians, pathologists, and neonatologists. In addition, the placenta is a highly complex organ system, and our fundamental understanding of the patterns of placental injury, including the timing and combined presence of multiple lesions, is still emerging (1).

The impact of placental dysfunction and placental injury on the fetus and newborn infant has become a topic of growing interest in neonatal research. However, the use of placental pathology in directing or influencing neonatal clinical management continues to be limited for a wide range of reasons, some of which are historical and thus easily overcome today. In this review, we will summarize the most recent literature linking placental function to neonatal outcomes, focusing on clinical placental pathology findings and the most common neonatal diagnoses that have been associated with placental dysfunction. We will discuss how recent technological advances in neonatal and perinatal medicine may allow us to make a paradigm shift, in which valuable information provided by the placenta could be used to guide neonatal management more effectively, and to ultimately enhance neonatal care in order to improve our patient outcomes. We propose new avenues of clinical management in which the placenta could serve as a diagnostic tool toward more personalized NICU management.

## Overview of recent literature

A recent literature search conducted by our group querying “placenta and neonatal outcome” resulted in 5,741 results since 1961 accessible through PubMed.gov (https://pubmed.ncbi.nlm.nih.gov). Totally, 4,703 are in the past 20 years (2003–2023), and 50% of these publications are from 2016 to 2023 alone. Thus, there has been a rapidly increasing interest in studying the placenta as it pertains to the neonate. The body of literature is quite diverse. While the bulk of studies investigate novel pathways by which the placenta may lead to or predict adverse neonatal outcomes, very few evaluate placental pathology as a diagnostic and management tool for neonatologists (2), including our previous publication in which we discussed the clinical applications of the placenta for neonatologists (3). Still to date, many of the more recent studies of placental pathology that evaluate “neonatal outcome” continue to focus on preterm birth and fetal growth restriction as the outcomes. Very few studies extend these findings to propose how the neonatologist can use placental information in caring for NICU patients—specifically in preventing or minimizing morbidities for which newer therapies and treatment strategies have since been developed.

Recent reports have linked specific lesions of the placenta to infant complications of high-risk pregnancies, including neonatal outcomes such as brain injury, heart and lung diseases (both acquired and congenital), and a wide range of diagnoses with long-term neurodevelopmental, cardiopulmonary, and metabolic sequelae. With technological advances over the years, novel structural, cellular, functional, and genetic targets have been explored, but these “pregnancy outcomes” have been largely limited to the immediate birth outcomes such as stillbirth, preterm birth, and low birth weight. Epidemiological studies led by neonatal investigators are more likely to include postnatal outcomes during the NICU course, such as the major complications of prematurity [e.g., bronchopulmonary dysplasia (BPD), necrotizing enterocolitis, and intraventricular hemorrhage, among others]. Collaboration with obstetric and pathology colleagues is critical to our understanding of how the placenta may drive these neonatal outcomes. In addition, important mediators between maternal complications of pregnancy, placental findings, and later childhood outcomes must be taken into consideration when unraveling these complex associations.

## Gross and histologic features of the placenta: relevance to neonatal management

The standard placental pathological examination conducted by experienced placental pathologists worldwide is a valuable, robust tool that has contributed significantly to our understanding of maternal and fetal health during pregnancy. The most widely used guidelines originated from the Amsterdam Placental Workshop Group, published in 2016 by Khong et al. (4), which established consensus with respect to both sampling of the placenta and criteria for major placental lesions. These definitions were recently updated in a report by Redline et al. (1) as criteria to outline four distinct domains of placental pathology, thereby facilitating classification of the so-called “patterns of placental injury” and providing a framework for understanding the developmental and pathologic processes linked to specific clinical outcomes. This reference serves as a practical resource that can be incorporated into routine neonatal management. Discussion with placental pathologists and obstetricians is essential for interpreting how the timing and presence of multiple lesions and their potential synergistic effects could have contributed to certain neonatal clinical presentations. These criteria and patterns have been leveraged over the years by various groups (5, 6) and provide significant data that the neonatologist can use to understand the intrauterine environment and exposures during fetal development, with implications for management of critically ill neonates.

**Figure 1:**
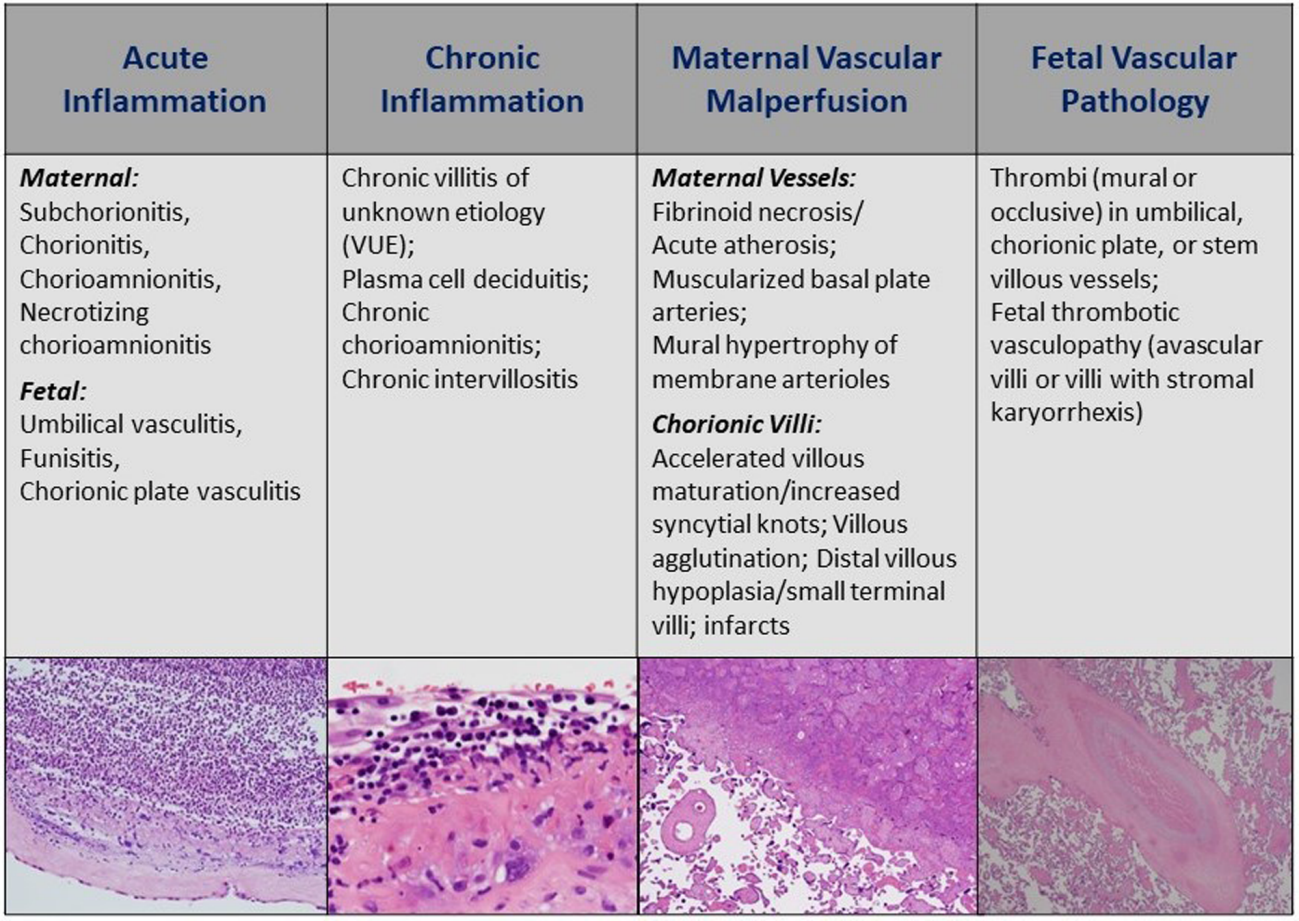
Four major histologic domains of the standard placental pathology exam. AI, acute inflammation; CI, chronic inflammation; MVM, maternal vascular malperfusion; FVP, fetal vascular pathology (also referred to as fetal vascular malperfusion or FVM). Histology images provided by Northwestern University Feinberg School of Medicine, Department of Pathology.

Figure 1 describes the four domains of histopathology with distinct lesions reported on standard pathology reports reviewed by clinicians. In terms of relevance to newborn infants and NICU patients, the presence of acute inflammatory (AI) lesions strongly suggests recent or active intrauterine infection by bacterial, viral, or fungal pathogens and should warrant sepsis evaluation and even empiric treatment for culture-negative sepsis in the newborn period (7). Chronic inflammatory (CI) lesions are less prominently reported but may suggest more long-standing placental injury and dysfunction early in pregnancy driven by immune-mediated processes between mother and fetus (8). An intriguing CI lesion is villitis of unknown etiology (VUE), which has been seen with viral pathologies and various degrees of fetal growth restriction thought to be mediated by immune rejection (9). The presence of maternal vascular malperfusion (MVM) is understood to occur very early in pregnancy—it is linked to abnormalities in trophoblast implantation and development, and failed maternal spiral artery remodeling that leads to abnormal villous development, compromised maternal-fetal gas and nutrient exchange, and chronic fetal hypoxia. MVM is commonly seen in cases of maternal preeclampsia and severe fetal growth restriction, and is of growing interest in the management of premature infants born through such exposures (10, 11). Finally, fetal vascular pathology (FVP) or malperfusion (FVM) indicates reduced or absent perfusion of the villous parenchyma by the fetus. Most common etiologies include umbilical cord obstruction leading to stasis, ischemia, and thrombosis. Other proposed causes include fetal cardiac insufficiency, hyperviscosity, and thrombophilias (12).

In addition to the above histologic domains, common gross pathologic lesions include placental weight, infarction, hemorrhage, and cord abnormalities, which can be readily identified at delivery. Alone or in conjunction with histologic lesions, these gross findings may be important in the immediate care of the critically ill neonate. For example, massive retroplacental/intraplacental hemorrhage and cord abnormalities, including hypercoiling and true knots, may prompt additional tests and monitoring for bleeding, thrombosis, or end-organ involvement not readily apparent on the newborn examination.

## Neonatal outcomes of perinatal origins: opportunities to leverage the placenta

While the patient population in the NICU is highly complex, there is a growing recognition that certain neonatal diagnoses arise from pathogenic processes that occur in the prenatal and perinatal periods of fetal development. For these diagnoses, the placenta may provide key insight into management approaches for which there are multiple pathways. In other words, more focused management plans could be tailored based on placental findings. In Table 1, we summarize the most recently published associations between placental gross and histologic lesions and common neonatal problems that have growing evidence of originating in the fetal stages and are considered a result of placental dysfunction (in traditional NICU presentation approach—by systems). A couple of the most prominent themes in our search of placental pathology and common NICU diagnoses are described below.

**Table 1:** Common conditions treated in the NICU (by systems) with described placental associations.

System/disease	Placental association(s)	Clinical management implications
Fluids, electrolytes, and nutrition		
Hypoglycemia	Placental syncytial knots, calcifications (13) Placental weight, placental malperfusion lesions (14)	May narrow differential diagnosis: placental insufficiency/injury vs. infectious etiologies, which would shift management toward or away from prolonged antibiotic treatments. May identify cause and predict course of management for hypoglycemia in the absence of small-for-gestational age birth weight.
Postnatal growth failure	Maternal and fetal inflammatory response (15, 16)	May distinguish constitutional growth and other causes of growth delays from inflammatory processes, leading to different approaches in feeding/nutrition support and long-term follow-up.
Cardiorespiratory problems		
Apnea/bradycardia	Methylxanthines readily pass placental barrier and may effect control of breathing mechanisms in infancy (17)	Normal placental pathology may rule out pathologic causes of apnea (i.e., intrauterine infection, neurologic), thus, facilitating/confirming diagnosis of apnea of prematurity.
Respiratory distress syndrome	Histologic chorioamnionitis with pathologic causes of surfactant deficiency (18, 19)	In preterm infants, normal placenta may facilitate diagnosis of noninfectious causes of hyaline membrane disease (i.e., delayed surfactant production) vs. sepsis/pneumonia.
Meconium aspiration syndrome	Columnar epithelial changes vs. meconium-laden macrophages differentially associated with adverse neonatal outcome; funisitis (20, 21)	Placental changes could inform the extent and timing of causes, and thus guide work-up and management approaches (e.g., treat for infection, work-up for perinatal depression, supportive respiratory care).
BPD	HCA and FIR (inconclusive in meta-analyses) (22) and decreased risk of BPD and death (23) MVM increased with BPD-associated pulmonary hypertension (10, 24) Chorionic plate vascularization (25) Chronic inflammatory lesions (26)	Could help differentiate certain endotypes of BPD, such as inflammatory versus vascular, which could inform response to specific treatments of which there are currently multiple approaches with untoward side effects (e.g., steroids, pulmonary vasodilators, diuretics, chronic mechanical ventilation).
Congenital heart diseases (coarctation, septal defects, tetralogy of Fallot, transposition of great arteries, hypoplastic left heart, double outlet RV)	Abnormal villous maturation (27), increased MVM lesions, lower placental weight, abnormal umbilical cord insertion, distal villous FVM, villous edema, amnion nodosum (28, 29), DNA methylation changes (30), MVM or FVM, abnormal umbilical cord coil, placental perfusion defects (31, 32)	Could help inform the etiologies of certain heart defects, as well as intrauterine conditions and events that may further compound complex care: timing of surgical interventions, associated morbidities, anticipated clinical course, counseling for prognosis and recurrence risk in future pregnancies.
CDH	Shallow placental implantation, diffuse chronic hypoxic patterns of placental injury, lesions of fetal vascular malperfusion, likely stasis-induced (33)	As above. Severity and persistence of pulmonary hypertension may be worse with evidence of placental vascular disease, thus informing management decisions regarding need for ECMO, timing of surgical repair, prognosis.
Gastrointestinal problems		
Gastroschisis/abdominal wall defects	Placental ischemia, MVM, suggesting that vascular events in second trimester cause disruption of fetal abdominal wall formation (34)	May provide additional information on extent and chronicity of intestinal injury involvement, which could guide decisions on surgical management, feeding, and risks for intestinal dysfunction.
NEC	Multiple placental lesions, including placental inflammatory and vascular lesions, is associated with higher risk for medical or surgical NEC (35); Massive perivillous fibrin deposition with FGR (36); Histologic chorio with preeclampsia (37)	May guide NICU management decisions regarding risk for NEC, and thus timing of initiation and advancement of enteral feedings.
Neurologic problems		
HIE	Review of 26 articles: FVM, umbilical cord pathology, inflammatory changes, MVM, placental weight (38); Hypercoiled cord, abnormal insertion (39); Chronic placental pathologies associated with more severe brain injury (40); Combined acute and chronic placental changes are common, underscoring the complex causal pathways of HIE (41)	Placental findings could inform the causes of HIE, of which there are multiple. For example, acute inflammatory changes of histologic chorio should prompt infectious work-up, including for meningitis. Fetal thrombotic vasculopathy may suggest vaso-occlusive causes and prompt more neurologic work-up and supportive management. MVM and VUE may suggest chronic placental insufficiency and thus more complex involvement of multiple organ systems and slower recovery.
Neonatal seizures/stroke	Both acute and chronic placental inflammation are common in perinatal stroke (42); FIR with vascular thrombotic problem (43)	May provide key information on timing and etiology of seizures, thus prompting specific work-up leading to targeted treatments, including antibiotics, thrombolytic therapies, genetic and hematologic work-up for recurrence risks.
IVH	Massive perivillous fibrin deposition with FGR (36); MVM in premature and SGA infants (44)	May identify infants at relatively higher risk for IVH, and influence early management decisions regarding surveillance and neuroprotection strategies in “small baby” protocols.
PVL	FIR (43); Chronic diffuse capsular deciduitis, decidual plasma cells, evidence of chronic but not acute chorioamnionitis (45)	Findings in premature infants may prompt more comprehensive early neuroimaging (MRI), early intervention services, and long-term neurodevelopment follow-up.
Infectious diseases		
Neonatal sepsis/pneumonia/meningitis	FIR (46); histologic chorioamnionitis (47); variable among many small studies, associations may be modified by antepartum prophylaxis and other clinical considerations (48)	Presence of FIR/HCA without positive blood culture may point toward partially treated infection, chronic infection, etc. prompting antibiotic treatment. Negative placental histology with positive neonatal sepsis work-up may suggest acute infection or false positive blood test in a newborn infant.
TORCH and other congenital infections	Toxoplasmosis: villous trophoblast cell apoptosis (49); Syphilis: Hydrops placentalis, funisitis, fetal vasculopathy, chronic villitis (50); Rubella: Obliteration of stem vessels, villous inflammation and sclerosis, trophoblastic necrosis (51); CMV: chronic villitis, CMV inclusions (52); HSV: Nonspecific findings (53)	See Yallapragada et al. (3)
COVID-19	Multiple lesions reported (54–56). No evidence of direct viral involvement or vertical transmission. Most recent studies report that there are no significant placental histopathologic changes that occur after dx of SARS-CoV2 infection in women during third trimester (57)	Current literature provides reassurance supporting minimal need for NICU surveillance or intervention, but long-term studies are still needed for guidance in pediatric follow-up.

CMV, cytomegalovirus; ECMO, extracorporeal membrane oxygenation; FIR, fetal inflammatory response; FVM, fetal vascular malperfusion; FGR, fetal growth restriction; HCA, histologic chorioamnionitis; HSV, herpes simplex virus; MVM, maternal vascular malperfusion; TORCH, toxoplasmosis, other, rubella, CMV, HSV; NICU, neonatal intensive care unit; BPD, bronchopulmonary dysplasia; CDH, congenital diaphragmatic hernia; NEC, necrotizing enterocolitis; HIE, hypoxic-ischemic encephalopathy; VUE, villitis of unknown etiology; IVH, intraventricular hemorrhage; PVL, periventricular leukomalacia; SGA, small for gestational age; RV, right ventricle.

### Neonatal hypoglycemia

There have been very few studies linking neonatal hypoglycemia and placental findings that could potentially enhance management. In a small case–control study, Natarajan et al. reported an association between placental syncytial knots [adjusted odds ratio (aOR) (95% CI), 2.9 (1.1–9.1); *P* = 0.04] and placental calcifications [aOR (95% CI), 3 (1.1–8.7); *P* = 0.03] as predictors of small for gestational age (SGA) birth weight, in which higher rates of in-hospital morbidities included perinatal asphyxia, respiratory distress, hypoglycemia, and feeding intolerance (13). Although these associations are relatively weak, they remind us that clinical observations of hypoglycemia in SGA infants could be due to chronic placental insufficiency in some cases, allowing neonatologists to tailor the management accordingly. This might mean using fewer tests to look for other causes of hypoglycemia and feeding intolerance, such as sepsis and hyperinsulinism. Recent findings from a study by Hutcheon et al. using latent profile analysis in a much larger cohort (26,007 births from Montreal, Canada, 2001–2009) suggest that placental weight and placental malperfusion lesions may serve as novel markers to identify infants at risk for hypoglycemia due to placental insufficiency, independent of the SGA proxy (14).

### Respiratory distress syndrome

The majority of early literature on placental histopathology and neonatal outcomes was in reference to histologic chorioamnionitis (HCA), which is a relatively common finding in spontaneous preterm birth. In recent studies and large meta-analyses, the link between HCA and neonatal outcome has been reported at most as modest (58), with the exception of respiratory distress syndrome (RDS) (18)—a primary problem of surfactant deficiency in the newborn period. In a large cohort study conducted in Australia, Lahra et al. found a protective effect of maternal and fetal inflammation on RDS (59). This may suggest multiple modalities of RDS beyond surfactant deficiency that need to be investigated in ongoing studies.

### Bronchopulmonary dysplasia

**Figure 2:**
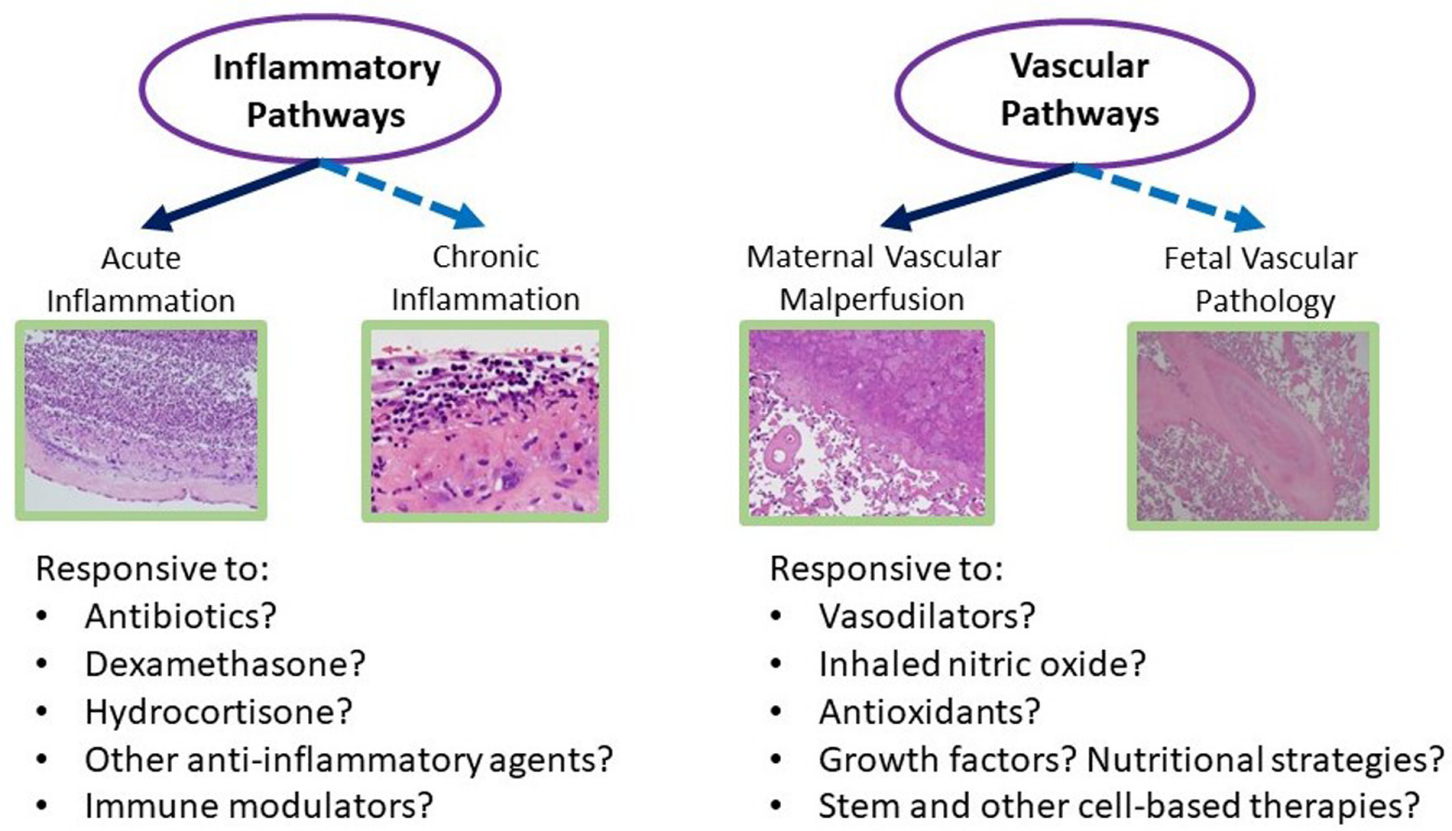
Placental pathways of lung disease: proposed schematic of how detailed review of placental histology along the different pathologic domains can guide NICU management of multifactorial diseases such as BPD. NICU, neonatal intensive care unit; BPD, bronchopulmonary dysplasia.

In our search, we found that long-standing reports linking placental inflammation to BPD are variable and appear to be refuted by several recent multicenter studies and meta-analyses. In contrast, more recent studies link placental vascular disease, specifically MVM, to sub-phenotypes of BPD-associated pulmonary hypertension (60). Inferring these “endotypes” and linking them to placental disease may have implications for neonatal management (see Figure 2) (61). Recent reports on the interaction between HCA and exposure to antenatal steroids to accelerate lung maturation and attenuate BPD suggest that infants born through intrauterine inflammation have been protected by antenatal steroids (62). Alternatively, in a recent analysis of postnatal corticosteroid response in neonates <32 weeks and their associated placental pathologies, a trend of better steroid response was observed in infants exposed to MVM (63). These and other studies provide evidence that response to certain postnatal therapies can be predicted by placental information (Figure 2).

### Congenital diaphragmatic hernia

Genetic variants account for 30% of congenital diaphragmatic hernia (CDH) cases that likely occur via a complex inheritance pattern influenced by *in utero* stressors through placental function and metabolism. Genetic variants likely confer susceptibility with epigenetic modifiers (e.g., nutritional factors, toxins, hypoxia) contributing to the subsequent phenotype (64). The role of vitamin A metabolites has been well-described in normal placental maintenance and embryologic lung development via the retinoic acid signaling pathway (65). Retinoic acid receptor responder proteins regulate antioxidant responses, angiogenesis, and anti-inflammatory processes. This signaling pathway and/or other functionally similar ones may contribute to the placental pathology that has been described in these patients. Placental phenotypes are suggestive of shallow placental implantation, lesions of stasis-associated FVM, and chronic patterns of hypoxia (33). Other work analyzing placental pathology of CDH patients also demonstrated increased placental fetal thrombotic vasculopathy (66). While a degree of pulmonary hypertension may be explained by primary defects in lung and pulmonary vascular development, there may be a yet undefined contribution from MVM (10). Further understanding of these placental factors may be helpful to prognosticate and potentially change the continued high mortality and morbidity in these patients.

### Congenital heart disease

A rapidly evolving area of neonatal research has focused on mechanisms of congenital heart disease (CHD) and placental dysfunction. The fetal heart placenta axis describes processes that regulate the development of the placenta, to both heart development and function (67, 68). Abnormal placental development may impact cardiac development, and the circulatory changes from congenital heart disease may result in placental insufficiency (69). Newborns with CHD have smaller placentas, which put them at risk for SGA. Lesions of FVM in the placenta have been associated with structural cardiac defects in the fetus (70). These studies provide compelling evidence that the placenta may play a causative role in the embryonic development of the heart, independent of, or interactively with, genetic predispositions and environmental exposures during pregnancy. However, there is still much to be investigated regarding the role of the placenta in the management of congenital heart defects. The most severe forms of CHD are rare, and the growing recognition of CHD subtypes makes our understanding of placental relationships even more challenging (71). Thus, studies that continue to use animal and *in vitro* models of placental-cardiac development are critical to our understanding of how and to what extent placental development influences fetal heart function (72, 73). Moreover, ongoing clinical research efforts to link placental findings to expanding longitudinal cohorts of CHD infants are critically needed to advance the field of neonatal-cardiac intensive care (74). As CHD is not a commonly recognized indication for placental autopsy and many subtypes/cases are not diagnosed prenatally or in the immediate postnatal period, efforts that reliably link placental features to CHD management will require well-designed, prospective studies across institutions and over many years.

### Hypoxic-ischemic encephalopathy

Studies of placental pathologic findings and hypoxic-ischemic encephalopathy (HIE) continue to be published. The studies on this topic appear to have increased in the past decade due to the implementation of therapeutic hypothermia in level 3 and 4 NICUs nationwide. The question of whether the placental pathology examination may be incorporated into the decision-making process to initiate therapeutic hypothermia in a newborn infant with HIE is important, as there is ongoing controversy over the level of HIE and the effectiveness of current clinical information to predict response to therapy (e.g., cord blood gases, Apgar scores, physical examination findings). Since infants require initiation of cooling within 6 h of life, the utility of the placental examination would either need to be expedited or proxies of placental histology developed (e.g., cord blood, neonatal blood, maternal blood). Given the proximity of these biospecimens to the placenta at birth, development of these proxies seems feasible and promising. The association between placental findings and neurodevelopmental outcome in neonates with HIE is less apparent in the era of therapeutic hypothermia, suggesting that neonatal intervention is effective in uncoupling the placental-perinatal events leading up to HIE, by attenuating the acute process of brain injury and improving long-term outcomes (75).

### Neonatal sepsis

Finally, the research and clinical attempts to use placental histologic features to guide management of neonatal sepsis (and rule out sepsis) continues to be highly controversial and not universally accepted. To our knowledge, there are no recent meta-analyses evaluating the literature on the use of the placenta in neonatal sepsis management. Rather, considerable research has focused on the appropriate timing, indications, and dosage of intrapartum antibiotics given to mothers in order to prevent neonatal sepsis and its comorbidities (76). Thus, intrapartum antibiotic exposure is likely an important mediator that confounds our observations between placental findings and neonatal sepsis. As such, there are no routine uses or published protocols for incorporation of the placental examination in ruling in or ruling out neonatal infections. A recent study by Wong and Khong found umbilical cord section diagnosis of funisitis to be reasonably accurate in cases with clinical chorioamnionitis, fever, and/or premature rupture of membranes. They propose changing current laboratory practice to rapid processing of umbilical cords ahead of the rest of the other placental sections in busy pathology departments (46). These changes seem feasible and warranted, given the evidence that the presence of funisitis or vasculitis can rule in sepsis and other diagnoses requiring immediate intervention.

## Current clinical limitations and research gaps

Despite the wealth of evidence accumulated over the years on associations between placental pathology and neonatal outcomes, why is the placenta not used more routinely in postnatal management of the neonate? At most large academic birth centers, standardized placental pathologic examination is routinely performed on a subset of all deliveries, based on published recommended guidelines. The criteria prompting examination, which have recently been updated (77), are historically and predominantly driven by maternal, fetal, and intrapartum indications (e.g., preeclampsia, clinical chorioamnionitis, intrauterine growth restriction, fetal distress, abruption) with neonatal indications limited to diagnoses requiring immediate care (i.e., hypoxic-ischemic encephalopathy, sepsis, meconium aspiration). Even among these acute perinatal problems, there is a lack of recommendations for how the placenta can be used to individualize care. Much needed also is consideration for how placental findings could be used to guide ongoing NICU management for infants with multifactorial conditions such as extreme prematurity (e.g., BPD, NEC, IVH), congenital heart, lung, and intestinal anomalies—for which factors such as intrauterine infection and placental vascular disease may contribute to or complicate one's clinical course (Table 1). For some institutions, driven by state laws or hospital policies, preterm birth of a specified gestational age cut-off and/or delivery resulting in NICU admission are indications for placental pathology examination. This approach may capture the majority of diagnoses listed above. However, there is a need for more education and research to streamline, prioritize, and focus the pathologist's examination as to specific findings that may provide a more precision medicine approach for NICU patients (78).

There are several perceived and actual limitations in current clinical practice that may account for this disconnect. The turnaround time for placental reports to reach the neonatologist is relatively longer than blood and other biospecimen tests performed in clinical laboratories. In addition, the necessary expertise in placental/perinatal pathology is often limited and only readily available in tertiary care centers. Thus, requesting placental pathology results in level 2 and non-academic community NICUs is often perceived as not practical or feasible. However, well-coordinated and carefully designed workflows and infrastructures, many of which have been adopted in recent years to accommodate and facilitate patient biospecimens for research, can be implemented in a wide variety of hospital settings to preserve placental tissues for clinical care as well. Detailed protocols have been published that allow for a wide range of studies to be performed on placental tissues (79–81). Even the most common and routine methods for biopsy and archive of placental tissues, such as formalin-fixed paraffin-embedded (FFPE) tissues, can now be used to study spatial transcriptomics of the placenta (82). If properly processed, these tissues once thought useful only for routine histology could eventually be used to map the human placenta and enhance our understanding of a wide range of neonatal diseases. The above research strategies require the availability and use of specialized newer reagents, optimal workflows, and laboratory conditions, which are feasible through dedicated integration of clinical and research teams. Institutional support and funding that sustain these collaborative efforts are critical factors for success.

## Leveraging the placenta at the bedside: conceptual models

**Figure 3:**
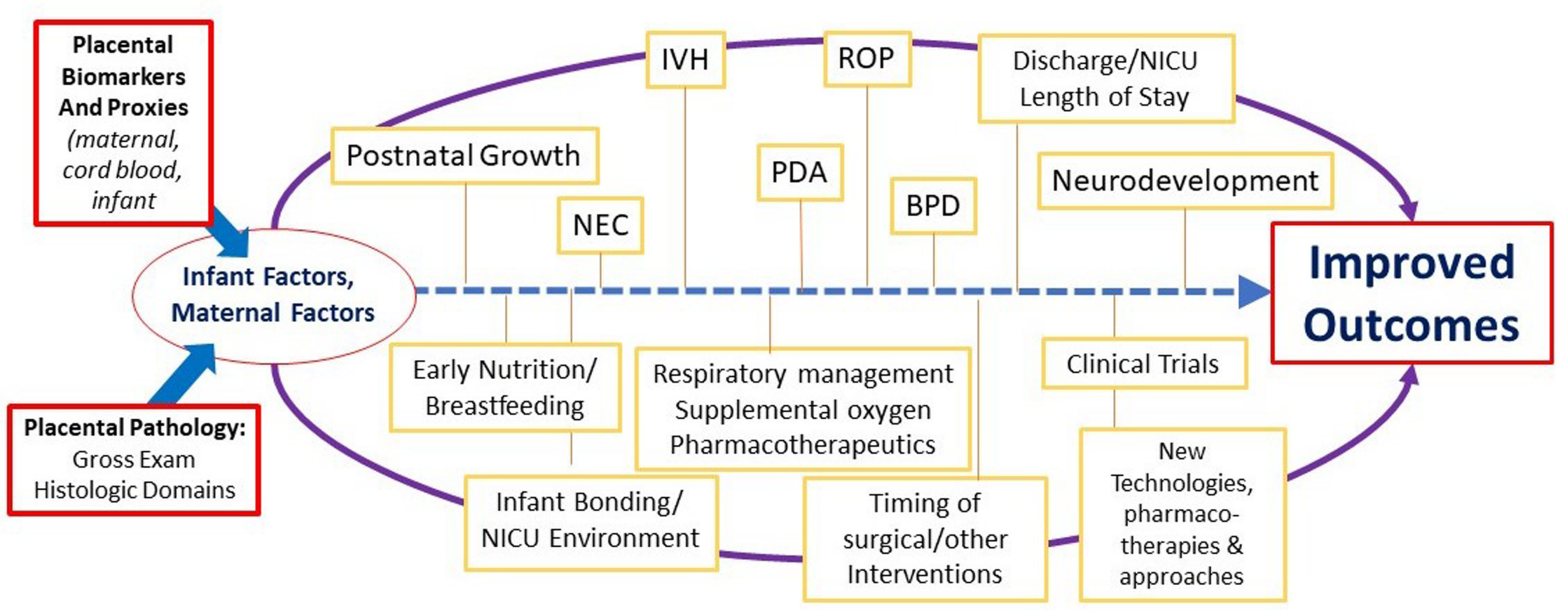
Overview of the potential role of the placenta in NICU management of preterm and low birth weight infants. BPD, bronchopulmonary dysplasia; IVH, intraventricular hemorrhage; NEC, necrotizing enterocolitis; NICU, neonatal intensive care unit; PDA, patent ductus arteriosus; ROP, retinopathy of prematurity.

Based on our ongoing research and work of others linking placental findings to neonatal outcomes, we have designed several conceptual models of how placental findings coupled with maternal data and the early perinatal course may help guide management of the critically ill infant in the later postnatal period for complex conditions such as those complicating preterm birth and low birth weight. Figure 2 illustrates such a conceptual model for BPD management, in which placental findings along pathways of acute and chronic inflammation may prompt decisions toward the use of anti-inflammatory strategies such as postnatal steroids, including type, timing, duration, and route of administration. Conversely, preterm infants with associated placental vascular pathology may be more responsive to pulmonary vasodilator therapies, and newer agents that promote pulmonary vascular growth. While these and other therapies require further investigation in larger and multicenter studies, the incorporation of placental findings into neonatal care throughout one's NICU course and in the development of newer therapies and clinical trials will help us further develop novel treatment strategies for a wide range of neonatal diseases (Figure 3).

## Need for placental biomarkers and proxies to inform NICU management

Placental examination findings and histologic lesions may serve as important biomarkers of *in utero* pathology affecting the critically ill neonate. The turnaround time for placental pathology reports should not obviate routine and comprehensive assessments, as many complications of prematurity and term infant diseases often do not require immediate readings, but within the first days/weeks of life. Certain decisions, such as the initiation and durations of antibiotics and therapeutic hypothermia, may require more immediate information, which could be prioritized in the clinical setting and coordinated with effective workflows and communication between services.

Gross placental pathology in many hospitals may take up to a week and histopathology reporting may take up to 3–6 weeks depending upon the patient volumes and other priorities of a given institution. Unfortunately, there are no well-established early rapid placental diagnostic tests that can be used to evaluate or identify specific lesions of placental injury before birth or point-of-care tests at delivery. Development of such technologies is an ongoing priority of our research efforts and the goal of many investigators committed to placental research (83, 84). Point-of-care tests during pregnancy or surrounding delivery that could distinguish between maternal and placental origins of disease, such as maternal circulating extracellular vesicle (EV) microRNAs, are promising but still in development (85, 86). Rapid, more automated diagnostics using placental imaging coupled with machine learning approaches are also emerging in the new era of artificial intelligence (87). These advances could enhance neonatal management by providing earlier risk assessment, anticipation of clinical course, and earlier initiation of certain NICU interventions upon delivery.

**Figure 4:**
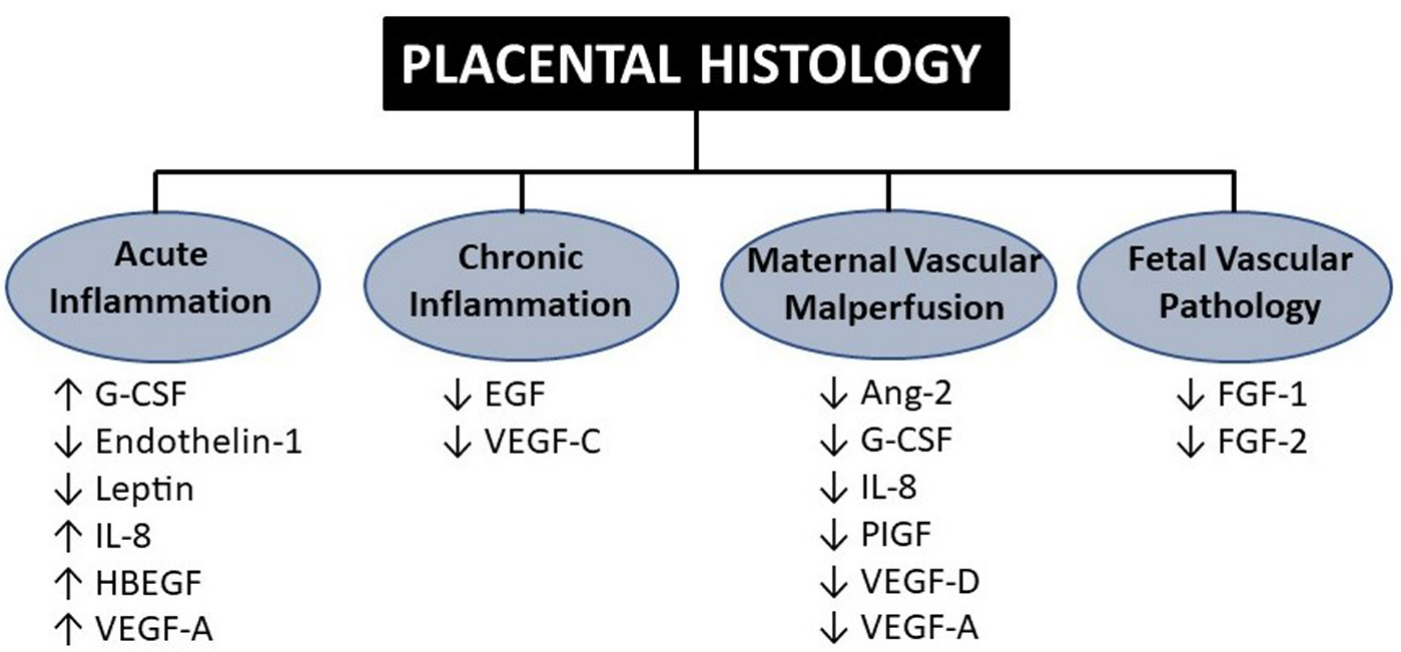
Cord blood biomarkers associated with the four domains of placental histology. Findings adapted from Mestan et al. in which 15 analytes were measured in cord blood plasma using Luminex multiplex immunoassay across four placental histologic domains. G-CSF, granulocyte colony-stimulating factor; IL, interleukin; VEGF, vascular endothelial growth factor; PLGF, placental growth factor; FGF, fibroblast growth factor (11).

Other strategies that could enhance the use of the placenta for neonatologists include the development of proxies and novel biomarkers that are tightly correlated with placental histology and detect placental dysfunction prior to or at birth. Maternal serum biomarkers of placental dysfunction are gradually being used in some centers to detect placental dysfunction due to maternal preeclampsia (88). In addition to the emerging use of maternal circulating sFlt-1/PIGF ratio to predict placental dysfunction-mediated preeclampsia, other potential proxies of placental function include levels of cytokines, chemokines, and growth factors circulating in cord blood at birth (11, 89–91). Studies measuring the biochemical milieu of the placenta have emerged over the years in attempts to link predictive markers of inflammation, oxidant stress, and other pathways to neonatal outcomes. Of particular interest are outcomes related to prematurity, such as brain injury (IVH, PVL), lung injury (BPD), and neurodevelopment (cerebral palsy). As detailed in Figure 4, biomarkers that delineate certain placental pathologic domains could be used to specifically address problems associated with systemic inflammation (IL-6 and IL-8) and dysregulated vascular endothelial growth (VEGF-A, placental growth factor).

## Future directions

The above review provides compelling evidence that the placenta is an important and valuable resource for NICU management. Recent breakthroughs and advances in technology provide even more potential approaches to leverage the placenta to enhance and improve neonatal care. Development of real-time early/rapid placental diagnostics such as placental imaging, including placental MRI during pregnancy and the use of digital image technologies linked with artificial intelligence and machine learning as mentioned above, may overcome limitations of regional availability of expertise and turnaround time for placental evaluation (87, 92). Development of more automated data entry may facilitate more rapid delivery of information to clinicians in NICU settings. The development of point-of-care testing is a recent and rapidly evolving approach to bedside diagnosis and more timely management of multiple problems in the NICU. These approaches could be applied to the evaluation of the placenta as well through closely related biomarkers in maternal blood/urine, cord blood, and postnatal biospecimens. Placental biopsies containing information on epigenomic, transcriptomic, and other multi-omics analyses with biological significance are rapidly being discovered (93, 94). Coupled with our current knowledge of placental histology and infant outcomes, the opportunities to advance neonatal care through the placenta are promising.
